# Local Population Structure and Seasonal Variability of *Borrelia garinii* Genotypes in *Ixodes ricinus* Ticks, Slovakia

**DOI:** 10.3390/ijerph17103607

**Published:** 2020-05-21

**Authors:** Zuzana Mtierová, Markéta Derdáková, Michal Chvostáč, Yuliya M. Didyk, Barbara Mangová, Veronika Rusňáková Tarageľová, Diana Selyemová, Alžbeta Šujanová, Radovan Václav

**Affiliations:** 1 Institute of Zoology, Slovak Academy of Sciences, Dúbravská cesta 9, 845 06 Bratislava, Slovakia; zuzana.mtierova@gmail.com (Z.M.); marketa.derdakova@gmail.com (M.D.); michal.chvostac@gmail.com (M.C.); yu.m.didyk@gmail.com (Y.M.D.); mangova.barbara@gmail.com (B.M.); veronika.taragelova@gmail.com (V.R.T.); Diana.Zelinkova@savba.sk (D.S.); alzbeta.vaskova@savba.sk (A.Š.); 2 Schmalhausen Institute of Zoology, NAS of Ukraine, B. Khmelnytskogo 15, 01030 Kyiv, Ukraine

**Keywords:** Lyme disease, *Turdus merula*, host ecology, geographic structuring, clonal complexes, ecological niche

## Abstract

Lyme disease (LD) is the most common tick-borne human disease in Europe, and *Borrelia garinii*, which is associated with avian reservoirs, is one of the most genetically diverse and widespread human pathogenic genospecies from the *B. burgdorferi* sensu lato (s.l.) complex. The clinical manifestations of LD are known to vary between regions and depend on the genetic strain even within *Borrelia* genospecies. It is thus of importance to explore the genetic diversity of such pathogenic borreliae for the wide range of host and ecological contexts. In this study, multilocus sequence typing (MLST) was employed to investigate the local population structure of *B. garinii* in *Ixodes ricinus* ticks. The study took place in a natural wetland in Slovakia, temporally encompassing spring and autumn bird migration periods as well as the breeding period of resident birds. In total, we examined 369 and 255 ticks collected from 78 birds and local vegetation, respectively. *B. burgdorferi* s.l. was detected in 43.4% (160/369) of ticks recovered from birds and in 26.3% (67/255) of questing ticks, respectively. Considering the ticks from bird hosts, the highest prevalence was found for single infections with *B. garinii* (22.5%). Infection intensity of *B. garinii* in bird-feeding ticks was significantly higher than that in questing ticks. We identified ten *B. garinii* sequence types (STs) occurring exclusively in bird-feeding ticks, two STs occurring exclusively in questing ticks, and one ST (ST 244) occurring in both ticks from birds and questing ticks. Four *B. garinii* STs were detected for the first time herein. With the exception of ST 93, we detected different STs in spring and summer for bird-feeding ticks. Our results are consistent with previous studies of the low geographic structuring of *B. garinii* genotypes. However, our study reveals some consistency in local ST occurrence and a geographic signal for one of the clonal complexes.

## 1. Introduction

Lyme disease (LD) is the most common tick-borne human disease in Europe [1,2]. *Borrelia burgdorferi* sensu lato (s.l.) currently represents a complex of 22 genospecies [3,4], ten of which are present in Europe: *B. burgdorferi* sensu stricto (s.s.), *B. garinii* [5], *B. afzelii* [6], *B. lusitaniae* [7], *B. valaisiana* [8], *B. bissettii* [9], *B. spielmanii* [10], *B. bavariensis* [11], *B. finlandensis* [12], and *B. turdi* [13]. In Europe, human LD is caused by various genospecies, but *B. afzelii* and *B. garinii* are by far the most widespread human pathogenic borreliae [14,15].

Concerning the two main human pathogenic *Borrelia* genospecies in Europe, there are important differences between *B. garinii* and *B. afzelii*. First, *B. garinii* appears to represent the genetically and phenotypically most polymorphic and heterogenous genospecies [9,16,17]. Second, despite the molecular polymorphism of *B. garinii*, the genetic differentiation between geographical areas is higher for *B. afzelii* than for *B. garinii* [18]. This observation is consistent with the idea that birds, as the main reservoir of *B. garinii*, are able to mix *B. garinii* strains between remote areas, while rodents, as the main reservoirs of *B. afzelii*, keep the spirochete subpopulations separate due to their limited movement [18]. Third, the prevalence of *B. afzelii* in questing ticks is usually higher than that of *B. garinii* (e.g., [19]), which is thought to reflect the higher densities of ticks supported by small mammals compared to birds [20]. Fourth, while the prevalence of *B. afzelii* in human LD patients is higher than that of *B. garinii*, the severity of Lyme disease is usually higher for the latter genospecies [21]. Additionally, the prevalence of sequence types (STs) detected in human patients for both genospecies is higher than expected based on the prevalence of these STs in questing ticks [21]. These findings indicate that different *Borrelia* strains can have different invasiveness and/or infectivity properties not only between but also within the genospecies [22,23].

It is accepted that genetic variability within the *B. burgdorferi* s.l. genospecies complex is associated with different clinical outcomes in LD patients [24] as well as with different reservoir hosts [25,26]. While *B. garinii* is mainly associated with neuroborreliosis, *B. afzelii* most often induces erythema migrans and acrodermatitis chronica atrophicans [15]. Yet, clinical features of infections with the same *Borrelia* genospecies can differ strikingly between regions, as shown by Cerar et al. [27]. Moreover, even though there are rather firm associations between *Borrelia* genospecies, tick vectors and vertebrate hosts, different reservoir hosts and tick species can be observed between regions even within the same *Borrelia* genospecies (see [28] for *B. garinii*.) Consequently, a certain *B. burgdorferi* s.l. strain can, from the pathogen’s perspective, perform well in one geographical or ecological setting, but this performance may not be representative, due to geographic differences in vector and/or reservoir capacity, across the strain’s distribution range or for the whole genospecies [29]. Thus, it is of importance for epidemiological and evolutionary studies to explore the genetic and phenotypic diversity of pathogenic borreliae for the wide range of host and ecological conditions.

The host community has been hypothesized as one of the main drivers of the intraspecific genetic diversity of *B. burgdorferi* s.l. via various mechanisms. According to one view, various hosts within a community can act as multiple ecological niches for a subset of strains of a species [30]. With respect to *B. garinii*, the concept of ecological niches can be rather complex for local avian communities involving migratory or transient bird species, because the niches fluctuate dynamically in space and time. Moreover, the majority of the European bird studies have focused on tick infestations at the time of autumn and spring migration, e.g., [31,32,33], whereas the role of songbirds in the transmission dynamics of borreliae at the breeding or wintering sites, as well as the role of resident birds, remain poorly investigated [34].

The aim of this study is to provide a first snapshot of the local population structure of *B. garinii* at a model study site in order to unravel the role of the avian community in the local circulation of a spirochete of human importance. Multilocus sequence typing (MLST) is used to reveal the local intraspecific diversity of *B. garinii* and its temporal within-season variation. Bird-feeding and questing *I. ricinus* ticks are examined over the period from spring to autumn avian migration in a natural wetland in Slovakia.

## 2. Materials and Methods

### 2.1. Study Area, Field Methods and Study Species

**Figure 1:**
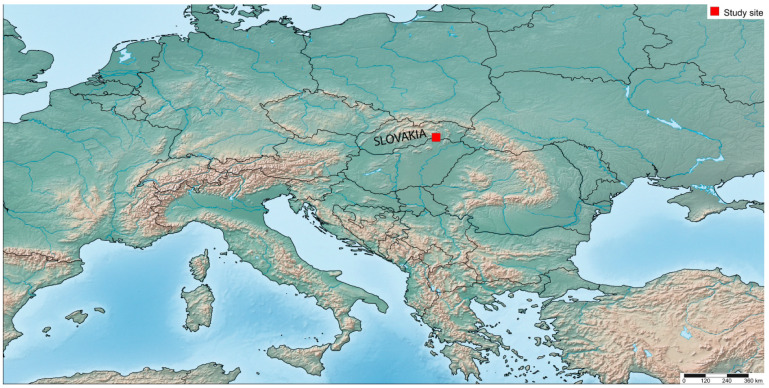
Study site location in south-east Slovakia. Map was plotted using SimpleMappr.

Ticks and birds were sampled in south-east Slovakia near the Drienovec village (48°10′11.303″ N; 17°4′2.255″ E; Figure 1). The study site (ca. 7.7 ha) is represented by a mosaic of woody wetland and forest-meadow ecotones at 190 m a.s.l. The Drienovec Bird Ringing Station has operated at the study site from 2006 and is involved in multiple bird-ringing schemes such as Constant Effort Sites (CES) and SE European Bird Migration Network (SEEN) [35]. Birds mist-netted in the framework of multiple ringing schemes were sampled for ticks in 2017. Birds were captured and banded under the permit of the Ministry of the Environment of the Slovak Republic No. 269/132/05-5.1_pil. Sampled birds were carefully examined for feeding ticks, and these were removed with fine forceps in tubes with 70% ethanol. Ring code, species and, if known, age and sex were recorded for each bird sampled. Ticks for each bird were stored in separate tubes. Questing ticks were collected at the study site by the standardized flagging method [36]. Both ticks from birds and questing ticks were collected during the same visits in 22–25 April, 24 June, 18–19 July and 19–21 October 2017. However, it was not possible to collect any questing tick in October due to rainy weather conditions. Totally, we collected 369 bird-feeding *Ixodes ricinus* ticks as well as 255, 23 and 14 questing *I. ricinus*, *Dermacentor marginatus* and *Haemaphysalis inermis*, respectively.

In addition to birds infested with ticks (Table 1), the following bird species (number of individuals) were examined for ticks with none found: *Cyanistes caeruleus* (42), *Carduelis carduelis* (34), *Sylvia curruca* (14), *Ficedula albicollis* (6), *Hirundo rustica* (4), *Emberiza citrinella* (4), *Poecile palustris* (4), *Pyrrhula pyrrhula* (3), *Regulus regulus* (3), *Remiz pendulinus* (3), *Sitta europaea* (2), *Linaria cannabina* (2), *Acrocephalus palustris* (2), *Aegithalos caudatus* (2), *Certhia familiaris* (2), *Phoenicurus ochruros* (1), *Buteo buteo* (1), *Dendrocopos major* (1), *Dryobates minor* (1), *Ficedula hypoleuca* (1), *Jynx torquilla* (1), *P. phoenicurus* (1), *Poecile montanus* (1), *Passer montanus* (1), *Phylloscopus sibilatrix* (1), *Serinus serinus* (1), and *Sylvia borin* (1).

*B. garinii* has the widest distribution of all the genospecies within the *B. burgdorferi* s.l. complex. This spirochete is not only found in wooded areas of Eurasia, where it is transmitted by *I. ricinus* and *I. persulcatus* [37], but also in marine areas, where it is transmitted by *I. uriae* in seabird colonies. Typical avian reservoirs of *B. garinii* comprise thrushes, pheasants and some seabirds [38,39,40,41]. In Asia, rodents may serve as reservoirs for *B. garinii* [42]. We only examine *I. ricinus* for borreliae in this study as this is the only confirmed vector of the spirochete in Europe [43]. Ticks were identified to species and life stages using available taxonomic keys [44].

### 2.2. DNA Extraction

A total of 369 bird-feeding and 255 questing *I. ricinus* ticks were examined using molecular techniques. The DNA from individual ticks was extracted by the alkaline-hydrolysis method [45] and eluted in 125 (larvae and unfed nymphs) and 250 (fed nymphs and adults) µL of MilliQ water [18]. 

DNA samples were stored at −20 °C. A 620-bp fragment of tick mitochondrial gene cytochrome *b* was amplified in each extracted sample to confirm the presence of the tick DNA [46]. Only positive samples were further analyzed for the presence of tick-borne agents.

### 2.3. PCR Amplification

HotStarTaq DNA Polymerase kit (Qiagen, Hilden, Germany) and 10 mM dNTP (Thermofisher, Dreieich, Germany) were used for all PCRs. PCR amplification was done in a BIO-RAD T100 Thermal Cycler (BIO-RAD, Hercules, CA, USA). Consequently, electrophoresis in 1.5% agarose gel and visualization on UV transilluminator Vilber-Lourmant (Sigma-Aldrich, St. Louis, MO, USA) was used for the detection of PCR products.

#### 2.3.1. PCR Screening of *Borrelia burgdorferi* s.l. DNA in Ixodes Ricinus

Each tick was tested for the presence of *B. burgdorferi* s.l. DNA. PCR amplification of a 222–255-bp fragment of *rrfA-rrlB* intergenic spacer was carried out using primers IGSA 5′-CGACCTTCTTCGCCTTAAAGC-3′ and IGSB 5′-AGCTCTTATTCGCTGATGGTA-3′ [47]. The PCR reaction was performed in a total reaction mixture of 25 µL. The PCR reaction mixture per each sample contained 2.5 µL of 10× PCR buffer, 1 µL of 25 mM MgCl_2_, 0.125 µL of HotStartTaq DNA polymerase (Qiagen, Hilden, Germany), 0.5 µL of both IGSA and IGSB primers (10 µM), 0.5 µL 10 mM dNTP (Thermofisher, Dreieich, Germany) and 14.875 µL of nuclease-free water (Promega, Madison, WI, USA). A quantity of 5 µL of tick DNA template was added to the reaction mixture. A touch-down PCR program was set following Derdáková et al. [47]. Positive samples were typed into Borrelial genospecies by Restriction Fragment Length Polymorphism (RFLP) analysis following Derdáková et al. [47]. A quantity of 13 µL of PCR product was mixed with 0.5 µL of Tru1I restriction enzyme (Fermentas, Thermo Scientific, Vilnius, Lithuainia) and 1.5 µL of buffer (Fermentas, Thermo Scientific, Vilnius, Lithuania). Digestion ran at 65 °C for 5 min. Electrophoretic separation was performed in the Origins system (Elchrom Scientific, Cham, Switzerland) using Spreadex EL300 mini gel (Elchrom Scientific, Cham, Switzerland) at 120 V for 150 min. After electrophoresis, the gel was stained with SYBR green (Sigma-Aldrich, St. Louis, MO, USA) for 45 min and visualized by UV transilluminator (Vilber-Lourmant; Sigma-Aldrich, St. Louis, MO, USA).

#### 2.3.2. PCR Amplification of 8 Housekeeping Genes (clpA, clpX, nifS, pepX, pyrG, recG, rplB, uvrA)

*Borrelia garinii*-positive samples were further analyzed using MLST according to protocol by Margos et al. [48]. PCR mixture for the first run of a nested PCR for all of the 8 housekeeping genes contained 2.5 µL of 10× PCR buffer, 1 µL of 25 mM MgCl_2_, 0.15 µL of HotStartTaq DNA Polymerase (Qiagen, Hilden, Germany), 1 µL of both outer forward and reverse primers (10 µM), 1 µL 10 mM dNTP (Thermofisher, Dreieich, Germany), 15.85 µL of nuclease-free water (Promega, Madison, WI, USA) and 2.5 µL of DNA template. The volume of PCR mixture in the second run of nested PCR was 30 µL per reaction and contained 3 µL of 10× PCR buffer, 1.5 µL of 25 mM MgCl_2_, 0.27 µL of HotStartTaq DNA Polymerase (Qiagen, Hilden, Germany), 1.5 µL of both inner forward and reverse primers (10 µM), 1.5 µL of 10 mM dNTP (Thermofisher, Dreieich, Germany) and 17.73 µL of nuclease-free water (Promega, Madison, WI, USA). A quantity of 3 µL of the PCR product amplified in the first run of nested PCR was added to the reaction mixture.

#### 2.3.3. Absolute Quantification of *B. garinii* by Droplet Digital PCR (ddPCR)

The number of exact DNA copies in selected *B. garinii*-positive samples from questing and bird-feeding *I. ricinus* was quantified using a QX200 Droplet digital PCR system (Bio-Rad, Hercules, CA, USA), amplifying a 75-bp fragment of *B. burgdorferi* 23S rRNA using primers Bb23Sf (5′-CGAGTCTTAAAAGGGCGATTTAGT-3′) and Bb23Sr (5′-GCTTCAGCCTGGCCATAAATAG-3′) with a TaqMan probe Bb23Sp-FAM (5′-AGATGTGGTAGACCCGAAGCCGAGTG) [49]. A quantity of 5 µL of DNA template was added to 10 µL of ddPCR Supermix for Probes (Bio-Rad, Hercules, CA, USA), 4.5 µL of each primer (900 nmol) and 1 µL of probe (250 nmol) to create droplets in droplet generator QX200 (Bio-Rad, Hercules, CA, USA). The droplets were run through a thermal cycler C1000 Touch^TM^ (Bio-Rad, Hercules, CA, USA) and read using a QX200 Droplet Reader (Bio-Rad, Hercules, CA, USA), which analyzed every droplet separately using the detection system set for FAM detection.

### 2.4. PCR Product Purification, Sanger Sequencing and MLST Analysis

Purification of PCR products intended for Sanger sequencing was done by NucleoSpin Gel and PCR Clean-up (MACHEREY-NAGEL, Düren, Germany). Sanger sequencing of all of the housekeeping genes was performed in Eurofins Genomics (Eurofins Genomics Germany GmbH, Ebersberg, Germany). Sequences obtained in this study were analyzed using the PubMLST database [50], with each allele receiving a number corresponding to an existing identical allele, or a new number in the case that the allele sequence was new to the database. Based on allelic profiles of 8 housekeeping genes, each sample was assigned an existing or new ST number [48]. GenBank accession numbers for the sequences of *B. garinii* alleles determined in this study are: MT371055 (*nifS* gene, PubMLST allele 231), MT371056 (*recG* gene, PubMLST allele 293), MT371057 (*nifS* gene, PubMLST allele 237), and MT371058 (*clpA* gene, PubMLST allele 302).

### 2.5. Phylogenetic Analysis

To examine clonal clustering of *B. garinii* STs obtained in this study with those from the PubMLST database, we used goeBURST algorithms with the PhyloViz 2 program [51,52]. The analysis was performed with all *B. garinii* isolates with a complete MLST profile deposited in the PubMLST database as of March 2020. In order to identify clonal clusters (CCs), goeBURST Distance analysis was conducted at the level of single-locus variants (SLVs). In turn, the goeBURST Full MST algorithm was used to generate the minimum-spanning tree. The tree was constructed at the maximal level of locus variants, i.e., level 8. This analysis provides a global perspective on relationships among STs, showing founders (central profiles) and closely related samples based on CCs. CCs from the MLST analysis and clades from the phylogenetic trees often reveal concordant results [53]. Country and tick source were used as auxiliary data for each isolate to aid visualizing accessory data in goeBURST analyses.

To investigate the phylogenetic relationships among *B. garinii* STs identified in this work and those deposited in the PubMLST database as of March 2020, we involved sequences for 147 *B. garinii* STs, whereas *B. burgdorferi* s.s. for ST 1 was used as an outgroup. Phylogenetic Maximum Likelihood analysis of concatenated sequences was conducted using the IQ-TREE web application [54]. The best-fit substitution model of sequence evolution was selected using the ModelFinder within the IQ-TREE platform [55]. The ModelFinder considers all traditional substitution models included in jModelTest and ProtTest [56,57], but also includes discrete Gamma (+G) [58] and FreeRate (+R) heterogeneity models [59], the latter representing a generalization of the discrete Gamma model. The best-fit model was selected with respect to Bayesian Information Criterion (BIC) scores. The branch support for the tree with the best substitution model was assessed using an aBayes test [60]. The resulting tree was unrooted, even though the outgroup taxon *B. burgdorferi* s.s. ST 1 was drawn at root. The tree was edited in iTOL v5 [61].

### 2.6. Statistical Analysis

In order to estimate the competence of bird hosts as reservoirs for borreliae [62], differences in *B. garinii* infection intensity with respect to two tick sources (bird-feeding vs. questing ticks) and two time periods (April vs. June–July) were analyzed with generalized linear models assuming negative binomial distribution. Since we obtained infection intensity data for two bird-feeding ticks from the same bird individual (i.e., the observations were not statistically independent), we conservatively removed the tick with higher infection intensity. A negative-binomial generalized linear model was run with the *MASS* package [63] in R [64]. Count data involving infection prevalences was analyzed using Fisher’s exact test with the *stats* package [64].

## 3. Results

### 3.1. B. burgdorferi s.l. in Bird-Feeding and Questing Ticks

In total, 369 feeding *I. ricinus* ticks were collected from birds over four sampling periods during April, June, July and October 2017. Overall, we sampled 504 birds of 41 species; 78 birds of 15 species were found to carry at least one blood-feeding tick. Of 369 bird-infesting ticks, 91 (24.66%), 277 (75.07%) and 1 (0.27%) ticks were in the larval, nymphal and adult stages, respectively. All of the ticks were examined for *B. burgdorferi* s.l. genospecies by RFLP analysis (Table 1).

In 29 *Borrelia*-positive samples, Sanger sequencing was used to confirm *Borrelia* genospecies determination by RFLP. These results confirmed genospecies determination by RFLP. In total, 160 (43.36%) ticks were *B. burgdorferi* s.l.-positive: 28/91 (30.77%), 132/277 (47.65%), and 0/1 (0%) of ticks in the larval, nymphal and adult stages, respectively.

Of 255 questing *I. ricinus* ticks, 16 (6.27%) were female adults, 21 (8.26%) were male adults, and 218 (85.49%) ticks were in the nymphal stage; no tick larva was collected by flagging. *B. burgdorferi* s.l. was detected in 67 (26.27%) ticks: 3/16 (18.75%) of female adults, 8/21 (38.10%) male adults, and 56/218 (25.69%) of nymphs. *B. afzelii* was a dominant genospecies for questing ticks (41/67, 61.19%), followed by *B. garinii* (11/67, 16.42%) and *B. valaisiana* (10/67, 14.93%). Four (5.97%) of the infected ticks showed mixed infection with *B. garinii* and *B. valaisiana*, and a mixed infection of *B. afzelii* and *B. valaisiana* was detected for one (1.49%) of the infected ticks.

### 3.2. B. garinii Infection Prevalence and Intensity in Bird-Feeding and Questing Ticks

**Figure 2:**
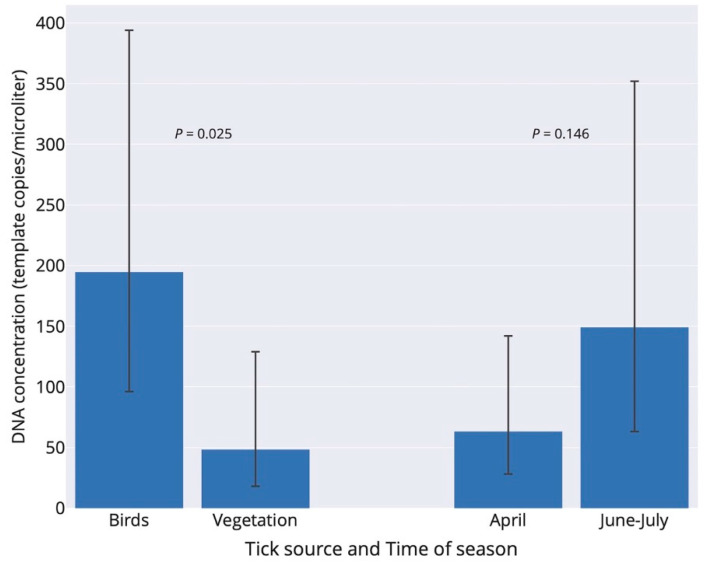
Infection intensity of *Borrelia garinii* in *Ixodes ricinus* ticks with respect to tick source (two bars in left) and time of season (two bars in right) in Slovakia. Infection intensities (number of copies/µL) refer to back-transformed estimates from the negative-binomial generalized linear model. Birds (*n* = 21 ticks) and vegetation (*n* = 11 ticks) denote bird-feeding and questing ticks, respectively. Bird-feeding ticks comprised 5 larvae and 16 nymphs, whereas questing ticks comprised 10 nymphs and 1 adult. The data for June and July were pooled. Errors are 95% confidence limits.

By pooling *I. ricinus* ticks with single and multiple *B. burgdorferi* s.l. infection that involved *B. garinii*, the prevalence of ticks infected with *B. garinii* was significantly higher in bird-feeding than questing ticks (106/160 vs. 15/67; Fisher exact test: *p* < 0.001). Moreover, *B. garinii* infection intensity, including only ticks with single *B. garinii* infection, was significantly higher in terms of the number of DNA copies in fed bird-feeding ticks compared to the intensity in questing ticks (negative binomial generalized linear model: tick source–df = 1, Chi-square = 5.00, *p* = 0.025; time of season–df = 1, Chi-square = 2.12, *p* = 0.146; Figure 2). The interaction effect of tick source and time of season was not significant (*p* = 0.625), and the interaction term was therefore not included in the final model.

### 3.3. Borrelia garinii STs

MLST analysis was conducted for 38 (45.8%) *B. garinii*-positive ticks collected from birds, involving the ticks for five bird species and four sampling periods. We were able to resolve ST profile for 18 ticks collected from 13 individual birds of four bird species (13, 2, 1, 1 and 1 ticks from *Turdus merula*, *Erithacus rubecula*, *Turdus philomelos*, *Parus major* and *Fringilla coelebs*, respectively) during three sampling periods (seven, six and five ticks from April, June and July, respectively). Identification of ST failed for six ticks due to the inability to amplify DNA templates, whereas mixed infection (different STs of the same genospecies) prevented identification of STs for 14 ticks. Of 18 bird-feeding ticks with ST profile, we detected 10 different *B. garinii* STs. Three of the STs were identified for the first time herein: STs 902, 929 and 933. The highest ST richness (7 STs) was found for ticks feeding on *T. merula*: STs 86, 93, 244, 246, 743, 902 and 933. Two STs were detected in ticks feeding on *E. rubecula* (STs 89 and 902). Single STs were found for ticks from *T. philomelos* (ST 87) and *F. coelebs* (ST 929). Considering two *T. merula* individuals for which we were able to resolve ST profile for multiple ticks, two ticks from one *T. merula* individual carried *B. garinii* of the same ST (ST 244), whereas five ticks from the second *T. merula* individual carried *B. garinii* of two STs (ST 246 and 902).

With respect to questing ticks, we conducted MLST analysis for all (11) ticks, showing *B. garinii* single infection. All of these ticks were collected during April (7) and June (4). ST was successfully established for six of the ticks (four from April and two from June); five ticks showed mixed infection, and ST determination was not possible for them. Totally, we have detected three STs for the *Borrelia* genospecies in questing ticks. Two of these STs—ST 172 and 903—were not detected in our sample of bird-feeding ticks. Conversely, only 1 of 10 STs detected in bird-feeding ticks was detected in questing ticks. In addition to three novel *B. garinii* STs detected in bird-feeding ticks in this study, we detected one novel ST for the *Borrelia* genospecies in questing ticks: ST 903. Overall, we detected 12 STs for bird-feeding and questing ticks at our study site over a single year.

**Figure 3:**
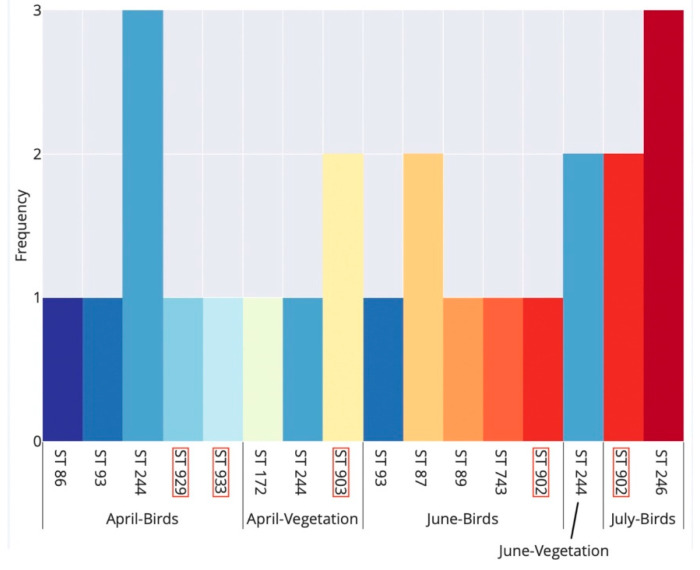
Seasonal occurrence of multi-locus sequence types (STs) of *Borrelia garinii* detected in bird-feeding and questing *Ixodes ricinus* ticks in Slovakia. Birds and vegetation denote bird-feeding and questing ticks, respectively. Each ST is denoted by a unique bar colour. ST labels with a red margin indicate novel STs detected for the first time in this study. Note that no ST was resolved for questing ticks in July.

Examining the phenology of *B. garinii* STs detected in bird-feeding and questing ticks, comparable ST richness was recorded during April (7 STs) and June (6 STs), whereas in July we only detected two STs (Figure 3).

There was a seasonal trend in the occurrence of *B. garinii* STs in bird-feeding ticks: STs 86, 929 and 933 were detected only in spring (April), while STs 87, 89, 246, 743 and 902 were detected only in summer (June–July). Only a single ST (ST 93) was detected in bird-feeding ticks both in spring and summer; ST 244 was detected in ticks during both spring and summer, but it only occurred in questing ticks in summer (Figure 3).

### 3.4. Phylogenetic and Geographic MLST Analysis

**Figure 4:**
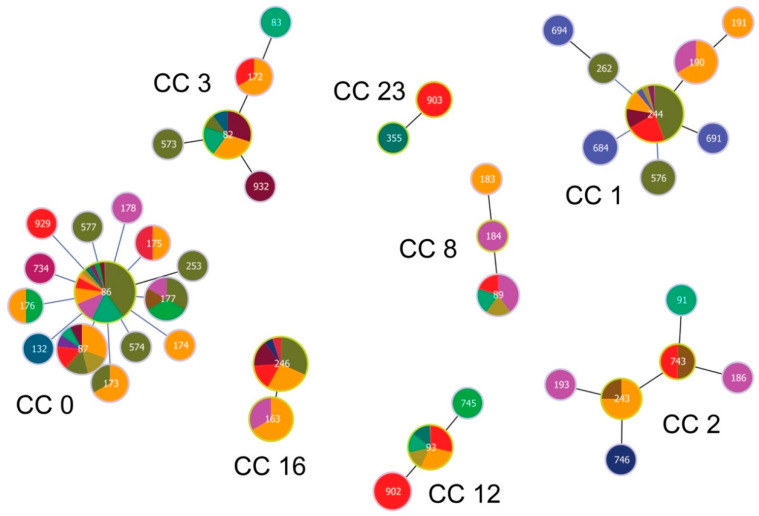
Population structure of *Borrelia garinii* multi-locus sequence types (STs) involving STs from Slovakia. Population structure for 147 STs deposited in the PubMLST database was constructed by the goeBURST algorithm with PhyloViz 2. Clonal complexes (CCs) were constructed at the level of single-locus variants (SLVs). ST 903 was not assigned into any CC and was classified as a singleton. ST nodes representing inferred CC founders are outlined in light-green color. Node size reflects the number of isolates for the ST. Black lines between nodes refer to links drawn without recourse to tiebreak rules, whereas blue lines show links based on tiebreak rule 1 (number of SLVs). Different colors and their areas within nodes reflect country and the number of isolates for the given ST and country. Red color denotes Slovakia; for country legend please see Figure 5.

GoeBURST analysis constructed at Single Locus Variant (SLV) level assigned 12 STs occurring at our study site into eight clonal complexes (CCs; Figure 4).

**Figure 5:**
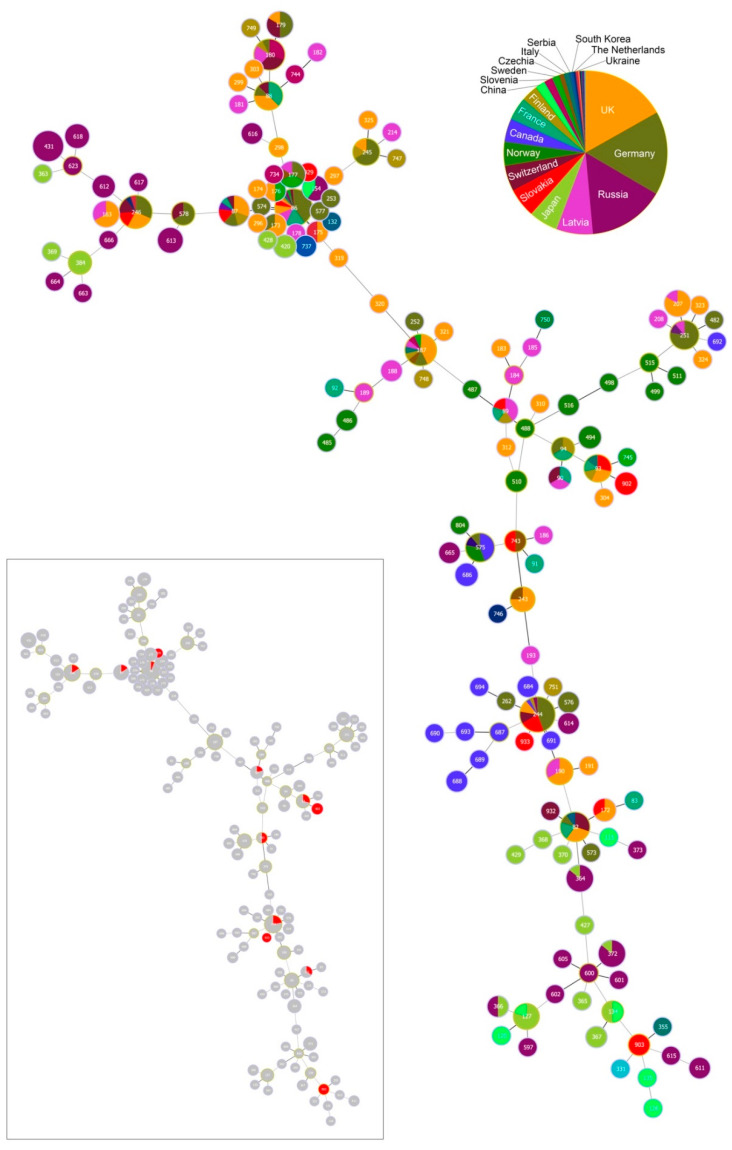
Minimum-spanning tree of *Borrelia garinii* multi-locus sequence types (STs). The tree was constructed for 147 STs deposited in the PubMLST database by the goeBURST Full MST algorithm with PhyloViz 2. The tree was constructed at the maximum level of locus variants (level 8). ST nodes representing inferred CC founders are outlined in light-green color. Node size reflects the number of isolates for the ST. The links having less differences between nodes are shown by darker grey color compared to links between more different nodes. Different colors and their area within nodes reflect country and the number of isolates for the given ST and country. The tree miniature highlights STs detected in Slovakia (red colour).

**Figure 6:**
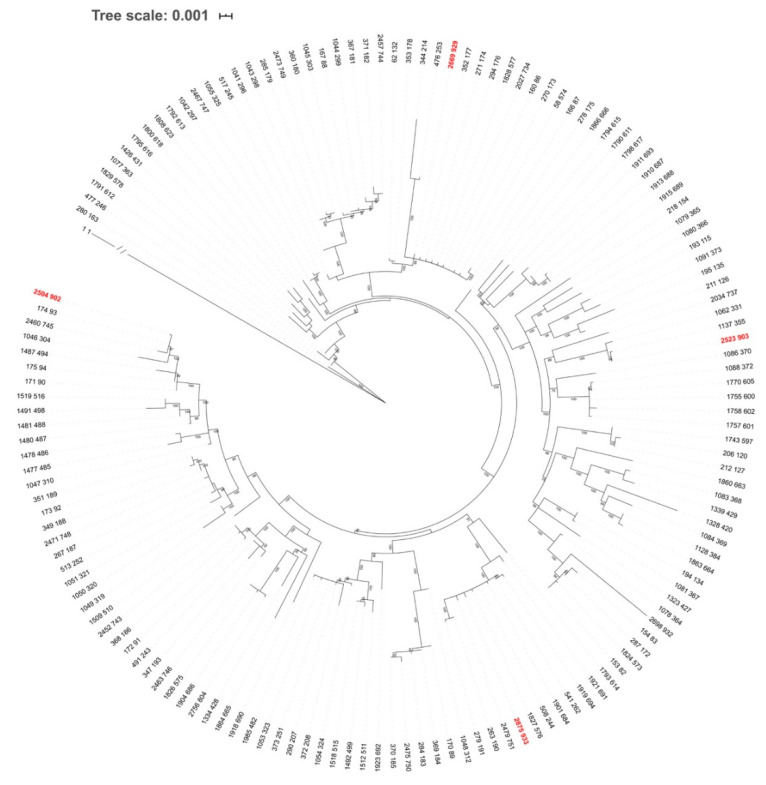
Unrooted ML tree of *Borrelia garinii* based on concatenated sequences of eight MLST genes. The tree involves sequences for 147 *B. garinii* sequence types (STs) deposited in the PubMLST database. Even though the tree is unrooted, *B. burgdorferi* s.s. (ST 1) was used as outgroup and was drawn at root. Branch length for *B. burgdorferi* s.s. is not scaled as indicated by the slashes. ML tree was constructed using IQ-TREE, based on the best-fit substitution model (GTR-F-R5) selected according to ModelFinder. The branch support was assessed using the aBayes test and is shown for branches with >70% support. Taxon labels are isolate and ST IDs according to the PubMLST database. STs detected for the first time in this study are marked in red.

One ST (ST 933) did not belong to any CC and was classified as a singleton. Inspecting the relationships among the twelve STs and those from the PubMLST database [50] reveals the occurrence of STs of diverse phylogenetic origin at our study site (Figure 5 and Figure 6)

The most frequently occurring STs at our study site belong to CC 0, 1, 12 and 16. While STs from CC 0, 1 and 16 tend to be phylogenetically older and show a cosmopolitan distribution, STs from CC 12, but also from CC 2, 3, 8 and 23 for the more sparsely occurring STs, are phylogenetically younger and show a stronger geographic signal (Figure 4; Table 2). In fact, CC 2 displays a particular geographic affinity to the study region, as the inferred founder *B. garinii* genotypes (ST 243 and 743) for this CC were almost entirely detected in ticks from Slovakia and Czechia (Figure 4; Table 2).

**Figure 7:**
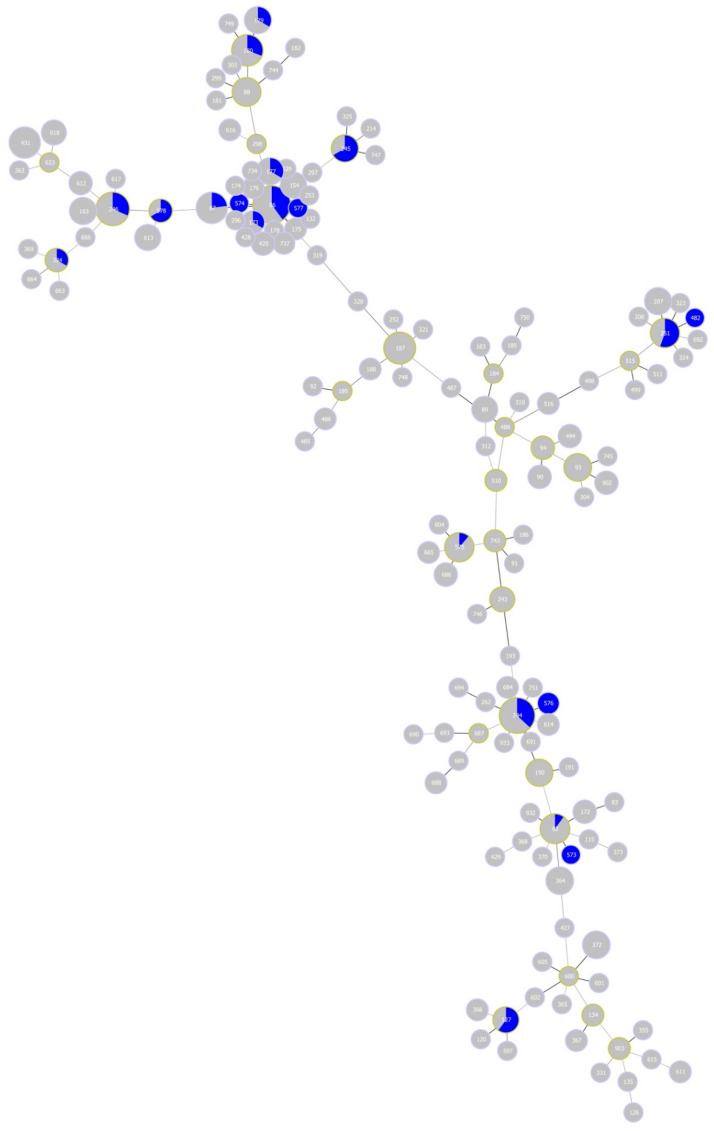
Minimum-spanning tree of *Borrelia garinii* multi-locus sequence types (STs). The tree highlights STs detected in samples from Lyme disease human patients (blue colour). Blue area within nodes reflects the number of isolates for the given ST.

Finally, *B. garinii* STs for half of the eight CCs associated with the study site, namely, CC 0, 1, 3 and 16, were previously detected in human tissue samples elsewhere (Figure 7). The vast majority (41/42) of these samples were obtained from Germany; one sample was recovered from the former Yugoslavia.

## 4. Discussion

In this study, intra-seasonal changes in the local population structure of *Borrelia garinii* spirochetes derived from bird-feeding and questing *Ixodes ricinus* ticks have been examined for the first time. Over the period of one year, spanning from spring to autumn bird migration, and covering the breeding period of resident birds and the period of tick seasonal activity, a local *I. ricinus* tick community was examined for intra-specific *B. garinii* genetic variability in a natural wetland in Slovakia.

Employing MLST, a relatively high richness of *B. garinii* STs was revealed considering a single site and season. Specifically, we identified 12 STs, one third of which were detected for the first time. In comparison, for countries with most thorough MLST data (Latvia, Germany, Russia, UK), 21 to 35 STs of *B. garinii* were detected at the country scale over multiple years [50]. Additionally, in a recent study on bird-feeding ticks conducted over multiple years at a European scale, authors were able to resolve 20 *B. garinii* STs (nine of which were new) for 11 countries [33]. Therefore, we believe that our study is sufficiently representative of a local richness of *B. garinii* genotypes using MLST.

We found a very small overlap in STs in questing and bird-feeding ticks; only one twelfth of STs (ST 244) were found in both tick sources. Such a result may be attributed to relatively low tick densities feeding on bird hosts, leading to relatively low prevalences of *B. garinii*-infected questing ticks (e.g., [19]) and a potential dilution of *B. garinii* STs in the local tick community. It is likely that the ratio in ST richness of bird-feeding to questing ticks would change with more intensive sampling of questing ticks or by examining ticks from unsampled bird species such as pheasants, *Phasianus colchicus* [65,66]. Yet, the small overlap in STs between the two tick sources could also be due to insufficiently low tick densities feeding on transient birds for the study site or due to lower infection prevalence and/or intensities of specific STs in local birds (c.f. [67]). Finally, differential invasiveness of *Borrelia* strains could explain differences in the prevalence of different *B. garinii* genotypes in vertebrate and tick hosts [22,23]. Regardless of the causes, our results suggest that quantification of the risk of *B. garinii* infection based solely on small-scale questing tick data is incomplete without information about the prevalence of different genotypes in local bird reservoirs. Specifically, this study indicates that the background ST richness based on local reservoir hosts can be much higher than ST richness in questing ticks. That is, even though the prevalence of some invasive *B. garinii* STs could be temporarily rare or absent in local questing ticks, the risk of human infection with these STs can be underestimated, given the STs regularly circulate in local bird reservoirs.

In addition to revealing largely different *B. garinii* ST communities for the two tick sources, we found a low seasonal overlap in STs for bird-feeding ticks; only a single ST (ST 93) was detected in bird-feeding ticks both in spring (April) and summer (June–July). It is important to note that we were not able to resolve any *B. garinii* ST for the autumn (bird migration) period. This could be related to the following two causes. First, in autumn, the prevalence of single *B. garinii* infection in *Borrelia*-positive bird-feeding ticks reached only 21.4% (3/14), whereas single *B. valaisiana* infection was detected for 50% (7/14) of bird-feeding ticks. Second, it was not possible to resolve ST for two *B. garinii*-positive ticks collected from *T. merula* in October, in one case due to multiple infection and in the other due to low infection intensity. It remains to be explored in further studies whether *B. valaisiana* indeed predominates during autumn in bird-feeding ticks at our study site and whether tick infection intensity with *B. garinii* is lower during autumn compared to spring and summer. Nevertheless, this study lends support to the appeal that the ecology of *B. garinii* should also be studied outside the period of spring and autumn bird migration [34]. In fact, our data suggest that infection intensity of *B. garinii* was comparable in bird-feeding ticks during spring and summer periods because the interaction effect of tick source and time of season on infection intensity was not significant. This finding thus challenges the view that in birds *B. garinii* infection is typically associated with migration (e.g., [26]).

We reveal a regular occurrence of mixed infection with different genotypes of *B. garinii* in bird-feeding and questing ticks. First, by means of MLST analysis, mixed infection with *B. garinii* genotypes was detected for 36.8% (14/38) and 45.5% (5/11) of bird-feeding and questing ticks, respectively. Second, being able to resolve STs for multiple ticks feeding on single *T. merula* individuals, we detected ticks with two STs (ST 246 and 902) in one of the birds. A high infection intensity of these ticks with *B. garinii* indicates that the bird was a reservoir of *B. garinii* of both STs. It is notable that the two STs belonged to different clonal complexes as identified by this study (Figure 4). Consequently, our study provides empiric evidence for the opportunity for gene recombination among different *Borrelia* strains within vertebrate and tick hosts [26,33,68,69]. We propose that the investigation of mixed infections involving different *B. garinii* genotypes would be fruitful for improving our understanding of the geographic variation in the occurrence of *B. garinii* of different invasiveness.

Based on our infection prevalence and intensity results, *T. merula* and *T. philomelos* appear to be the most important passerine reservoirs of *B. garinii* at our study site. While *B. garinii* was also detected in *Parus major*, *Erithacus rubecula* and *Fringilla coelebs*, infection in these species was either of low intensity (*Erithacus rubecula* and *Fringilla coelebs*) or prevalence (*Parus major*). Our study thus accords with previous results on the importance of thrushes in *B. garinii* occurrence from Slovakia and other European countries [34,40,41]. Nonetheless, our study stresses the epidemiological importance of a single thrush species, *T. merula*, which was by far most important in terms of species richness of *B. garinii* STs associated with this species as well as its infestation intensity by *I. ricinus* ticks. We suggest that this bird species is an ideal model for further research on *B. garinii* ecology even in the context of bird migration. The ring-recovery data for Europe show that the species’ populations from Scandinavia move for wintering to Britain and Ireland, and birds from Central Europe, including the birds from the study population, move to western Europe, Iberia and Italy, whereas Central Europe can in the same time serve as the wintering quarter for some eastern populations [70,71]. Moreover, the migration patterns of *T. merula* appear to be age- and sex-dependent, with females and young birds more likely to migrate than adult males. Consequently, there is a strong opportunity to exploit *T. merula*, but also *P. major*, by *I. ricinus* over the whole period of its seasonal activity, but the likelihood of infection with different *B. garinii* genotypes can vary considerably depending on the bird’s age and sex as well as on the weather conditions during winter. Finally, out of 12 STs detected at our study site, four STs (86, 87, 244 and 246) were previously isolated from samples of human patients with LD [50]. The ST 244 appears to be of the highest epidemiological importance at our study site because it was the only ST that was detected both in bird-feeding and questing ticks. Moreover, STs 86 and 244 belong to the most widespread STs, and their occurrence was detected in several continents [33,39]. Even though ST 244 was only detected in LD human patients from Germany, this may not reflect the ST’s true occurrence across Europe owing to absent or insufficient MLST data for human LD patients outside Germany. Such data are badly needed from more countries if we are to advance our understanding on the epidemiological risk factors for the most severe form of LD, namely, neuroborreliosis.

## 5. Conclusions

Our results are in accordance with previous studies on low geographic structuring of *B. garinii* genotypes across its distribution range [33,39,72]. However, our study also implies that it can be premature to generalize these results. First, the research on *B. garinii* ecology is mostly based on migrating birds [34] and, therefore, relies on methodologically biased data. Given that researchers investigate STs mainly for migrating birds, low geographic structuring of *B. garinii* genotypes is unsurprising. Second, by studying seasonal variation in the population structure of *B. garinii* STs, our study reveals that certain STs, such as ST 93, occur consistently in local bird-feeding ticks over spring and summer. Additionally, phylogenetic analysis showed that some of the genotypes, such as STs 243 and 743, appear to display a geographic signal and form inferred founder *B. garinii* genotypes for one of the clonal complexes. Therefore, some degree of geographical structuring may still occur for *B. garinii* genotypes in Europe [73]. In order to test this idea, however, it is essential to obtain the data at the appropriate temporal and spatial scale, namely, the wintering and breeding periods/quarters. Finally, we suggest that improved geographic coverage of MLST data involving human patients and avian hosts can turn out to be particularly revealing about the geographical structuring of *B. garinii* genotypes, the geographical origin of key bird reservoirs, and the geographical risk of infection with different strains of this genospecies.

## Figures and Tables

**Table 1:** Prevalence of *Borrelia burgdorferi* s.l. in *Ixodes ricinus* ticks feeding on birds at a study site in Slovakia, 2017. *L* and *N* refer to larvae and nymphs, respectively. Restriction Fragment Length Polymorphism (RFLP) was used to diagnose the genospecies of borreliae. Since ticks from the same birds were stored in single tubes, the possibility of sample contamination cannot be excluded, and the prevalences reported should be considered as maximum infection rates.

Bird Species	No. Birds Sampled	No. Birds Infested with Ticks	No. Birds with Infected Ticks	No. Ticks	No. (%) *B. burgdorferi* s.l. Infected Ticks	No. *B. garinii* Infected Ticks (L/N)	No. *B. valaisiana* Infected Ticks (L/N)	No. *B. garinii* + *B. valaisiana* Infected Ticks (L/N)	No. *B. afzelii* Infected Ticks (L/N)	No. *B. lusitaniae* Infected Ticks (L/N)	No. *B. spielmanii* Infected Ticks (L/N)	No. *B. garinii*/*B.b* +? Infected Ticks (L/N)	No. *B. valaisiana* +? Infected Ticks (L/N)	No. *B. afzelii* + *B. garinii* Infected Ticks (L/N)
*Turdus merula*	26	25	21	248	135 (54.44)	67 (13/54)	39 (5/34)	18 (3/15)	3 (0/3)	1 (0/1)	0	1 (0/1)	3 (1/2)	3 (0/3)
*Erithacus rubecula*	78	20	4	41	5 (12.20)	2 (0/2)	0	0	2 (0/2)	0	1 (1/0)	0	0	0
*C. coccothraustes*	16	8	2	22	2 (9.09)	0	1 (0/1)	0	1 (0/1)	0	0	0	0	0
*Parus major*	35	8	1	12	1 (8.33)	1 (0/1)	0	0	0	0	0	0	0	0
*Turdus philomelos*	4	4	1	16	12 (75.00)	11 (5/6)	0	1 (0/1)	0	0	0	0	0	0
*Garrulus glandarius*	3	2	0	11	0	0	0	0	0	0	0	0	0	0
*Luscinia megarhynchos*	6	2	0	4	0	0	0	0	0	0	0	0	0	0
*C. chloris*	10	2	1	2	1 (50.0)	0	0	0	1 (0/1)	0	0	0	0	0
*Fringilla coelebs*	8	1	1	5	3 (60.0)	2 (0/2)	0	0	1 (0/1)	0	0	0	0	0
*Phylloscopus collybita*	15	1	0	1	0	0	0	0	0	0	0	0	0	0
*Phylloscopus trochilus*	7	1	0	1	0	0	0	0	0	0	0	0	0	0
*Sylvia atricapilla*	109	1	0	1	0	0	0	0	0	0	0	0	0	0
*Sylvia communis*	3	1	0	1	0	0	0	0	0	0	0	0	0	0
*T. troglodytes*	12	1	0	3	0	0	0	0	0	0	0	0	0	0
*Prunella modularis*	31	1	1	1	1 (100)	0	0	0	1 (0/1)	0	0	0	0	0
Total	363	78	32	369	160 (43.36)	83	40	19	9	1	1	1	3	3

**Table 2:** Frequency of *Borrelia garinii* multi-locus STs detected in Slovakia and other countries based on the PubMLST database [50]. The source of *B. garinii* involved ticks and human tissue samples of Lyme disease patients. Tick source is split into bird-feeding (tick bird) and questing (tick veg.) ticks for Slovakia. Asterisk denotes a sample from Slovakia unrelated to this study.

	Slovakia		Austria	Canada	Czechia	Finland	France	Germany		Italy	Latvia	The Netherlands	Russia	Sweden	Switzerland	UK	Ukraine	Former Yugoslavia
ST	Tick Bird	Tick veg.	Tick	Tick	Tick	Tick	Tick	Tick	Human	Tick	Tick	Tick	Tick	Tick	Tick	Tick	Tick	Human
86	1	1 *	1	0	0	1	6	0	14	1	4	0	1	1	1	3	0	0
87	2	0	0	0	0	2	1	0	2	0	0	0	0	0	1	4	0	1
89	1	0	0	0	0	1	1	0	0	0	2	0	0	0	0	0	0	0
93	2	0	0	0	0	1	1	0	0	1	0	0	0	0	0	2	0	0
172	0	1	0	0	0	0	0	0	0	0	0	0	0	0	0	2	0	0
244	3	3	0	1	0	1	0	2	10	0	0	0	1	0	3	3	0	0
246	3	0	0	0	0	0	0	0	6	0	0	1	0	0	3	5	1	0
743	1	0	0	0	1	0	0	0	0	0	0	0	0	0	0	0	0	0
**902**	3	0	0	0	0	0	0	0	0	0	0	0	0	0	0	0	0	0
**903**	0	2	0	0	0	0	0	0	0	0	0	0	0	0	0	0	0	0
**929**	1	0	0	0	0	0	0	0	0	0	0	0	0	0	0	0	0	0
**933**	1	0	0	0	0	0	0	0	0	0	0	0	0	0	0	0	0	0

STs detected for the first time in this study are in bold letters.
